# The effects of differing anticoagulant regimes on blood quality after cell salvage in coronary artery bypass grafting (CABG): a pilot study

**DOI:** 10.1186/s13019-023-02246-w

**Published:** 2023-04-08

**Authors:** Willem Boer, Mathias van Tornout, Maarten Brusseleers, Maarten Strauven, Pieter de Vooght, Margot Vander Laenen, Eric Hoste, Philippe G. Jorens

**Affiliations:** 1 Department of Anesthesiology, Intensive Care Medicine, Emergency Medicine and Pain Medicine, Ziekenhuis Oost Limburg ZOL, Genk, Belgium; 2 Department of Anesthesiology and Intensive Care Medicine, AZ Sint-Jan Brugge-Oostende AV, Brugge, Belgium; 3 Intensive Care Unit, Department of Internal Medicine and Pediatrics, Ghent University Hospital, Ghent University, Ghent, Belgium; 4 Research Foundation Flanders (FWO), Brussels, Belgium; 5 Department of Critical Care Medicine, Antwerp University Hospital, University of Antwerp, LEMP, Edegem, Belgium

**Keywords:** Cell salvage, Citrate, Heparin, Blood quality, Inflammation

## Abstract

**Background:**

Cell salvage reduces allogenic blood transfusion requirements in surgery. We present a pilot study exploring the impact of anticoagulant choice, citrate or heparin, on the quality of cell salvaged blood in adults undergoing coronary artery bypass grafting (CABG).

**Materials and methods:**

Elective on pump CABG patients were randomly allocated to citrate or heparin anticoagulation. We measured red blood cell characteristics and inflammation in both the blood collection reservoir and the washed red blood cell concentrate. Postoperatively, the level of biomarkers and the coagulation profile in the peripheral blood as well as the transfusion requirements of allogenic blood products were studied.

**Results:**

Thirty eight patients were included, 19 in the citrate group and 19 in the heparin group. Baseline characteristics were similar. In the washed red blood cell concentrate, Mean Hb (g/dl) and Ht (%) were lower in the citrate group [Hb: 18.1 g/dL (SD 1.3) vs. 21.1 (1.6), *p* < 0.001; Ht: 59.9% (54.7–60.9) vs. 63.7% (62.3–64.8); *p* < 0.001]; Mean corpuscular volume (MCV, μm 3) was higher [99.1fL (9.4) vs. 88 (4.2), *p* < 0.001] and mean corpuscular hemoglobin concentration (MCHC, g/dl) lower in the citrate group [31.9 g/dl (29.6–32.4) vs. 33.6 (33.1–34.0) *p* < 0.001]. Thrombocyte count (1000/μl) was higher in the citrate group [31.0 (26.0–77.0) vs. 13.0 (10.0–39.0); *p* = 0.006]. There were no differences in the requirement for allogenic blood products’ transfusion (intraoperatively and postoperatively) or in the coagulation parameters after washed red blood cell concentrate infusion. Higher IL-10 was found in the citrate group in the blood collection reservoir, higher neutrophil-derived myeloperoxidase (MPO) in the heparin group after washed red blood cell concentrate infusion.

**Conclusion:**

Though red blood cells in washed red blood cell concentrate were more swollen and diluted in the citrate group with more residual thrombocytes, published quality guidelines were met in both groups. Our pilot study suggests that differences in inflammatory markers in the blood collection reservoir and after infusion of washed red blood cell concentrate indicate a possible pro-inflammatory effect of heparin compared to citrate. A larger study is warranted to confirm these results and their possible clinical consequences.

*Trial registration* ClinicalTrials.gov: NCT02674906. Registered 5 February 2016.

**Supplementary Information:**

The online version contains supplementary material available at 10.1186/s13019-023-02246-w.

## Background

Allogenic blood transfusion, a necessary tool in managing perioperative blood loss, has several adverse effects. Perioperative allogenic blood transfusion, even when limited in volume, is associated with postoperative morbidity, increased length of stay and so cost, and mortality in both general and cardiac surgery [1, 2]. Cell salvage (CS) can reduce perioperative allogenic blood transfusion needs during cardiac surgery [3, 4]. Anticoagulation for CS can be achieved with either citrate or heparinized saline [5, 6]. Because of their properties and differing mechanisms of action, these anticoagulants differ in their impact on acid–base balance, cellular energy supply, calcium homeostasis, inflammation and oxidative stress [7, 8]. Cytosolic calcium (Ca^2+^) has an important signaling function mediated by releasing various signaling mediators from intracellular granules of activated blood platelets, polymorphonuclear cells, monocytes and macrophages. By modifying Ca^2+^ availability, citrate can exert a modulating effect on inflammatory signaling. Glucose and citrate, key components of citrate solutions, can also serve as energy sources for red blood cells [7, 8]. Heparin has an elaborate binding profile and can have both pro- and anti-inflammatory effects depending on its binding site, concentration and environment [7, 9–11].

Blood collected in the cell saver blood collection reservoir (BCR) can be used to study the effects of heparin vs. citrate directly on inflammatory parameters in red blood cells collected from the surgical field. To our knowledge, only one clinical study by Mortelmans et al. compared the effects of these anticoagulants in CS [12]. In this pilot study we aimed to study the effects of citrate and heparin anticoagulant regimens on quality of washed red blood cell concentrate (WRBC), and at various time points on inflammatory parameters and variables of hemolysis and coagulation, in adult patients undergoing on-pump coronary artery bypass graft surgery (CABG).

## Methods

This single center pilot study (NCT02674906) was performed in the operating room and the mixed medical-surgical ICU of Ziekenhuis Oost Limburg (ZOL), Genk, Belgium, a 805-bed non-university teaching hospital. This study was approved by the local ethics committee and written informed consent was obtained prior to surgery from the patient or legal representative. After consent, patients were randomized preoperatively either to a citrate anticoagulant group or a heparin anticoagulant group for cell salvage. During CABG, the perfusionist and anaesthesiologist were unblinded for the anticoagulation regimen used. Blinding was complete for the patient, as well as personnel in the intensive care unit (ICU) and ward.

Adult patients undergoing elective on-pump CABG were considered eligible for inclusion. Elective surgery was defined as planned at least 24 h before surgery. Off-pump CABG, urgent procedures, use of vasoactive medication prior to surgery, infection treated with antimicrobial therapy, chronic inflammatory disease, immune suppressive drug treatment, active neoplasia, renal replacement therapy, or use of extracorporeal membrane oxygenation (ECMO) were exclusion criteria. In addition, we excluded patients who had massive intraoperative bleeding that could not be safely managed while collecting study data. Patients could be excluded at any time if the inclusion criteria were no longer met or when exclusion criteria appeared. Patients were randomized using randomly generated treatment allocations within sealed opaque envelopes.

CS is a procedure to collect blood lost in the surgical field for reuse. Blood is collected in cell saver blood collection reservoir (BCR), where it is mixed with an anticoagulant, either citrate or heparinized saline. The collected blood-anticoagulant mixture is processed by filtering, after which it is drawn into a centrifuge. Isotonic saline solution is added to the centrifuge bowl as washing fluid. The centrifugal procedure separates red blood cells, which are denser and are propelled against the outer wall of the bowl, while less dense plasma moves towards the centre of the bowl where it is deposited in a waste bag. Waste products, including white blood cells, platelets, plasma, anticoagulant, fat, clotting factors, and free plasma haemoglobin are collected in the waste fluid. The washed red blood cell concentrate (WRBC) is collected in a separate bag.

Anaesthesia, cardiopulmonary bypass and cell salvage procedures were all executed according to the standard protocol used in our institution (see Additional file 1: Supplement 1). Fixed administration rates of anticoagulant to the cell saver circuit could potentially lead to clot formation in case of more than moderate blood loss and therefore BCR and its filter were primed with a larger volume of anticoagulant (300 ml, compared to 150–200 ml). During the cell salvage procedure measures were therefore taken to keep the anticoagulant to blood ratio (ATBR) as constant as possible. Cell salvage volumes (total fluid volume collected in BCR, incubation time, waste fluid and washing fluid used) and intraoperative diuresis were noted. Both type and volumes of fluids administered by the anaesthetist and perfusionist intraoperatively were tracked as well as the volume of red blood cells that were washed.

Washed red blood cell concentrate (WRBC) was transfused per-operatively or immediately post-operatively. If there was ongoing blood loss, the standard hospital protocol was applied to manage this; if there was necessity for allogenic blood transfusion, this was administered irrespective of the type of anticoagulant used (the ICU ward and the intensivist were blinded to the anticoagulant group).

The standard protocol left transfusion at the discretion of the board certified anesthesiologist and/or ICU staff member; a Hb < 8 g/dl in the peripheral blood is in our protocol considered the threshold for allogenic blood transfusion in this type of patients.

We collected baseline characteristics of patients before CABG. We measured effects of the specific anticoagulation protocol on quality of WRBC, and at various time points inflammatory parameters and variables of hemolysis and coagulation.

On one hand, the quality of WRBC was analysed by measuring hemoglobin (Hb), hematocrit (Ht), mean corpuscular volume (MCV), mean corpuscular hemoglobin (MCH), mean corpuscular hemoglobin concentration (MCHC), red cell distribution width (RDW), free hemoglobin (fHb), iron, thrombocytes, white blood cell (WBC) count and differentiation in WRBC compared to Hb and Hct in the BCR. Inflammatory parameters were analysed by measuring levels of interleukin (IL)-6, IL-8, IL-10 and myeloperoxidase (MPO) at baseline, in the BCR (in the blood-anti-coagulant mixture before processing) and in peripheral blood of the patient (taken from the arterial catheter in place) at two time points; Firstly, when the patient was successfully weaned from cardiopulmonary bypass, protamine had been given and when the WRBC had not yet been transfused. Secondly, in the ICU, 2–3 h post transfusion of WRBC, but before extubation or allogenic blood transfusion.

The impact after transfusion of WRBC was evaluated, apart from measuring inflammatory parameters, by measuring fHB, iron, transferrin, ferritin, haptoglobin, hepcidin, prothrombin time (PT), activated partial thromboplastin time (aPTT), international normalized ratio (INR), rotational thromboelastometry (ROTEM) in peripheral blood of the patient (taken from the arterial catheter), at the same two time points before and after transfusion of WRBC. Furthermore, blood loss in thoracic drains and transfusion requirements over the first 24 postoperative hours were compared between groups.

For determining the levels of interleukins and MPO, validated and commercially available assays were used: IL-6: Electrochemiluminescence immunoassay, (Elecsys Il-6 on Cobas e801); IL-8: human IL-8 ELISA kit (cat.no. KHC0081), Thermo Fischer Scientific; IL-10: human IL-10 ELISA kit (cat. No BMS215-2) Thermo Fischer Scientific; MPO: Myeloperoxidase ELISA kit (ref: KT-890) Epitope Diagnostics, inc. All assays were performed according to the manufacturers’ instructions, with internal quality controls and participation to external quality control programs.

Statistics were performed using SPSS^®^ Statistics version 28 (IBM). Normally distributed values were reported as the mean (SD), and non-normally distributed values were reported as the median (25–75th percentile). Because of the limited sample size, normality was determined by the Shapiro-Wilks test. Independent samples t-test or the Mann–Whitney U test were used as appropriate. The Paired Samples *t* Test or the Wilcoxon signed-rank test were used as appropriate to analyse values per patient. Chi^2^ was used for comparison of categorical values. A double-sided *p* value of less than 0.05 was considered statistically significant.

A sample size of 34 achieves 80% power to detect a difference of 2 g/dl in Hb, a primary outcome parameter, using a two-tailed two sample t-test at a 0.05 level of significance. Equal sized groups and a standard deviation of 2 was assumed.

## Results

Over a 3 month study period, a total of 38 patients were included, 19 patients in each group. There were no significant differences in baseline characteristics, including fasting lipid profiles, between the groups, apart from a higher lymphocyte count in the citrate exposed patients (of citrate group) (Table 1, Panel A) (laboratory values see Additional file 1: Supplement 2). Furthermore, only 2/19 (11%) patients in both groups were taking aspirin on the day of surgery (*p* = 1.000). In all other patients, antiplatelet therapy had been ceased. There were no differences in ICU length of stay (citrate group: median (25–75%) 3 (2–4) days; heparin group 3 (2–5) days, *p* = 0.300), hospital length of stay (citrate group: median (25–75%) 9 (8–11) days; heparin group 8 (7–10.5) days, *p* = 0.257) or mechanical ventilation (citrate group: median (25–75%) 8 (7–11) hours, heparin group 8 (7–13) hours, *p* = 0.665). There was no in-hospital mortality in the citrate group, one of 19 patients died in the ICU in the heparin group, due to massive middle cerebral artery stroke (5.3% *p* = 0.311).

**Table 1:** Panel A. Baseline Characteristics as mean (SD) or median (25–75%)

Parameter	All (n = 38)	Citrate (n = 19)	Heparin (n = 19)	*p*-value
*Panel A. Baseline*				
Age	64.2 (10.7)	62.6 (10.8)	65.8 (10.6)	0.362
Gender	M 31 (82%) F 7 (18%)	M 14 (74%) F5 (26%)	M 17 (89%) F2 (11%)	0.209
EUROSCORE II (%)	0.84 (0.65–1.86)	0.83 (0.60–1.28)	0.82 (0.71–2.46)	0.506
BMI	30.5 (5.3)	30.4 (5.6)	30.5 (5.1)	0.984
CABG ECC time (min)	142 (27)	135 (29)	150 (22)	0.080
CABG clamp time (min)	118 (29)	116 (30)	121 (29)	0.590
reperfusion time (min)	14 (11–23)	14 (9–17)	19(12–24)	0.221
*Panel B. Cell saver BCR*				
Hb (g/dL)	9.5 (8.0–10.6)	9.9 (7.1–10.7)	9.4 (8.5–10.8)	0.443
Hct (%)	26.5 (24.2–31.7)	29.8 (23.1–36.1)	26.4 (24.8–31.2)	0.977
IL-6 (pg/ml)	3969.00 (2022.50–7004.50)	3848 (1422.75–7073.25)	3994 (2569.00–6741.00)	0.730
IL-8 (pg/ml)	412,01 (175.97–596.00)	417.10 (168,35–681.35)	412.01 (216.82–558.64)	0.822
IL-10 (pg/ml)	163.85 (74.28–266.94)	203.70 (108.98–411.42)	108.10 (60.00–176.71)	**0.013**
MPO (ng/ml)	870.0 (281.7)	871.9 (301.8)	868.2 (269.5)	0.969
*Panel C. Washed RBC concentrate*				
Hb (g/dl)	19.6 (2.1)	18.1 (1.3)	21.1 (1.6)	**< .001**
Ht (%)	61.2 (56.6–64.2)	59.9 (54.7–60.9)	63.7 (62.3–64.8)	**< .001**
RBC (*10^6^/ml)	6.8 (5.9–7.2)	6.0 (5.4–6.3)	7.2 (7.0–7.4)	**< .001**
MCV (fL)	93.9 (8.9)	99.1 (9.4)	88.7 (4.2)	**< .001**
MCH (pg)	30.3 (1.5)	30.6 (1.4)	29.9 (1.5)	0.172
MCHC (g/dl)	33.0 (31.8–33.7)	31.9 (29.6–32.4)	33.6 (33.1–34.0)	**< 0.001**
RDW (%)	13.9 (13.6–15.0)	14.5 (13.7–15.5)	13.7 (13.2–14.8)	**0.032**
Thrombocytes (*1000/ml)	26.5 (12.0–41.8)	31.0 (26.0–77.0)	13.0 (10.0–39.0)	**0.006**
FHb (mg/dL)	158 (118–217)	156 (118–218)	160 (109–202)	0.908
iron (μg/dL)	24.0 (20.8–27.0)	22.0 (19.0–27.0)	24.0 (22.0–29.0)	0.258
WBC count (*1000/ml)	12.9 (9.7–19.0)	14.3 (9.7–22.4)	12.1 (7.5–16.7)	0.297
Neutrophils (%)	91.0 (86.0–95.0)	86.0 (85.0–91.0)	95.0 (90.0–97.0)	**< 0.001**
Lymphocytes (%)	6.0 (2.75–10.25)	10.0 (6.0–12.0)	4.0 (2.0–6.0)	**< 0.001**
Monocytes (%)	1.0 (0.0–1.0)	1.0 (1.0–2.0)	1.0 (0.0–1.0)	**0.014**
Eosinopils (%)	1.0 (0.0–1.0)	1.0 (1.0–2.0)	1.0 (0.0–1.0)	**0.043**
Basophiles (%)	0.0 (0.0–0.0)	0.0 (0.0–0.0)	0.0 (0.0–1.0)	0.563
Neutrophils (*1000/ml)	12.0 (8.1–17.4)	13.0 (9.2–20.5)	11.7 (6.3–15.9)	0.603
Lymphocytes(*1000/ml)	1.0 (0.4–1.6)	1.3 (1.1–2.4)	0.4 (0.2–0.7)	**< 0.001**
Monocytes (*1000/ml)	0.10 (0.08–0.30)	0.2 (0.1–0.3)	0.1 (0.0–0.1)	**0.014**
Eosinophils (*1000/ml)	0.1 (0.1–0.2)	0.1 (0.1–0.2)	0.1 (0.0–0.1)	**0.006**
Basophiles (*1000/ml)	0.0 (0.0–0.1)	0.0 (0.0–0.0)	0.0 (0.0–0.1)	0.435

Euroscore is a validated score to evaluate the risk of cardiac surgery (Nashef et al. EuroSCORE II. Eur J Cardio-thorac. 2012) [13]. BCR values were reported as mean (SD) or median (25–75%). Panel C. Values for washed RBC concentrate were reported as mean (SD) or median (25–75%)

Bold value < than 0.05 i.e. statistically significant

### The quality of WRBC

WRBC from patients in the citrate group had lower Hb, Ht and RBC count compared to heparin exposed patients (Table 1, Panel C). In addition, they had a higher mean corpuscular volume (MCV) and red blood cell distribution with (RDW), with a lower mean corpuscular hemoglobin concentration. Thrombocyte count in the WRBC samples was higher in the citrate group [31.0 (26.0–77.0) vs 13.0 (10.0–39.0); *p* = 0.006]. There were no differences between fHb and iron in the washed RBC concentrate.

WBC counts did not differ between groups in WRBC. WBC differentiation showed a higher count and percentage for lymphocytes, monocytes and eosinophils in the citrate group (see Table 1, Panel C) and a higher percentage (but not absolute count) of neutrophils in the heparin group.

### Inflammatory parameters

In the BCR, despite no difference at baseline, the anti-inflammatory marker IL-10 was significantly higher in the citrate group [203.70 (108.98–411.42) vs 108.10 (60.00–176.71) *p* = 0.013]. There were no significant differences in the collecting bowl for the other inflammatory markers, Hb and Ht (see Table 1, Panel B).

Before WRBC transfusion, there were no significant differences in inflammatory parameters between the groups (see Additional file 1: Supplement 2). After WRBC transfusion (see Table 2), MPO was higher in the heparin group than in the citrate group [92.5 (83.5–117.3) vs 129.9 (112.5–187.9), *p* = 0.019]. There were no differences in other inflammatory parameters.

**Table 2:** Results in patients after WRBC transfusion, reported as mean (SD) or median (25–75%)

Parameter	All (n = 38)	Citrate (n = 19)	Heparin (n = 19)	*p*-value
Fhb (mg/dL)	12.0 (9.0–18.3)	12.0 (9.0–18.0)	11.0 (9.0–19.0)	0.729
Iron (μg/dL)	89.5 (65.3–117.8)	76.0 (66.0–110.0)	94.0 (63.0–120.0)	0.470
Transferrin (g/l)	1.3 (1.1–1.6)	1.6 (1.1–1.6)	1.3 (1.2–1.5)	0.471
Ferritin (μg/l)	224.7 (150.86)	193.70 (136.39)	255.73 (161.72)	0.209
Hepcidin (ng/ml)	20.1 (11.5–29.2)	18.0 (9.4–21.8)	25.4 (12.6–29.7)	0.189
Haptoglobin (g/l)	0.49 (0.35–0.69)	0.49 (0.25–0.70)	0.52 (0.37–0.71)	0.722
INR	1.35 (1.27–1.45)	1.30 (1.26–1.42)	1.39 (1.27–1.46)	0.212
Pt (%)	62.0 (55.8–68.0)	66.0 (57.0–68.0)	59.0 (55.00–68.0)	0.234
aPTT (s)	33.4 (30.8–35.1)	33.7 (30.5–35.0)	33.1 (31.3–35.3)	0.885
IL-6 (pg/ml)	481.0 (365.0–757.8)	493.0 (200.0–697.0)	461.0 (378.3–896.8)	0.379
IL-8 (pg/ml)	17.7 (15.6–35.8)	19.8 (15.6–31.4)	15.7 (15.6–38.2)	0.606
IL-10 (pg/ml)	31.0 (18.4–56.3)	35.2 (19.3–52.4)	27.8 (12.7–71.8)	0.461
MPO (ng/ml)	114.1 (91.3–168.8)	92.5 (83.5–117.3)	129.9 (112.5–187.9)	**0.019**

Bold value < than 0.05 i.e. statistically significant

### The impact after transfusion of WRBC

Free Hb, iron, ferritin, haptoglobin and hepcidin did not differ between the groups, both before and after WRBC transfusion. There was no difference in aPTT, PT, INR and ROTEM analysis before and after WRBC transfusion between groups. Both intra-operatively and post-operatively there was no difference between the groups in the need for transfusion of allogenic blood products (Packed Cells, Thrombocytes, Fresh Frozen Plasma) and total blood volume lost during the first 24 post-operative hours was comparable (see Table 3).

**Table 3:** Intra- and post-operative allogenic blood product tranfusion and blood loss

Parameter	General	Citrate	Heparin	*p*-value
Packed cells transfused intraoperatively (units)	0.13 (0.41)	0.05 (0.23)	0.21 (0.54)	0.583
Thrombocytes transfused intraoperatively (pools)	0.08 (0.27)	0.05 (0.23)	0.11 (0.31)	0.795
FFP transfused intraoperatively (units)	0.11 (0.39)	0.11 (0.46)	0.11 (0.31)	0.817
PC transfused 24 h postoperatively (units)	0.58 (0.98)	0.37 (0.76)	0.79 (1.13)	0.284
Thrombocytes transfused 24 h postoperatively (pools)	0.13 (0.41)	0.11 (0.46)	0.16 (0.38)	0.624
FFP transfused 24 h postoperatively (units)	0.37 (0.88)	0.11 (0.46)	0.63 (1.12)	0.258
Blood loss in 24 h (ml)	931 (424)	902 (410)	959 (448)	0.603

For readability, volumes of tranfusion products are given as mean (SD). *p*-values are calculated using the appropriate non-parametric tests

### Cell salvage volumes

There were no significant differences between study groups in total fluid volume collected in BCR, ATBR, incubation time, waste fluid and washing fluid. Although the volume of WRBC was higher in the citrate group this difference was not significant. The significant difference in Ht in WRBC between groups (see Table 1, Panel C) was therefore offset by the higher volume of WRBC in the citrate group, resulting in no significant difference in the product of Ht and the volume of WBRC. The red blood cell volumes washed twice did not differ between groups [citrate 45 (61) ml, heparin 67 (53) ml, *p* = 0.172] and were small. The number of patients per group receiving twice washed red blood cells did not differ significantly [10/19 (53%) in the citrate group, 15/19 (79%) in the heparin group, *p* = 0.087].

No significant differences were noted in fluid volume or type, administered before, during and after cardiopulmonary bypass. Intraoperative diuresis was not significantly different between the groups. The total heparin dose during cardiopulmonary bypass was not significantly different between the groups (Citrate group: mean heparin dose: 32,361 (11,776) IU, heparin group: 36,711 (13,087), *p* = 0.296). There were no early surgical revisions in either study group (apart from 1 sternal revision in the heparin group, 4 days after surgery).

## Discussion

In this trial comparing the effects of differing anticoagulant regimens (citrate or heparin) on cell salvage in adult patients undergoing elective pump CABG, small but relevant differences were observed between the two groups. WRBC had a significantly lower hematocrit and higher MCV and a larger residual thrombocyte count in the citrate group. There were also differences in WBC differentiation between groups. In the collecting bowl (before the cell salvage washing procedure but after treatment with anti-coagulant) IL-10 was significantly higher in the citrate group. After WRBC transfusion in the patients, there were no differences in interleukin blood levels between the groups, but MPO was significantly higher in patients in the heparin group. Both intra-operatively and post-operatively, this did not result in differences in transfusion of allogenic blood products or blood loss. Washed red blood cell concentrates in both groups met the quality guidelines published by the American Association of Blood Banks [14].

We believe the differences in MCV and MCHC can be attributed to a number of factors. ACD-A is hypotonic (400–440 mOsm/kg, sodium content 214–234 mEq/l) and acidic (pH 4.5–5.5). Both have been implicated in increased MCV, the latter especially when ACD-A concentrations are relatively high [15]. Increased MCV and RDW values in the citrate group reflect the findings by Mortelmans et al. [12]. MCHC differences can be attributed to the changes in MCV as MCH values do not differ significantly. In contrast to the Mortelmans study [12], in which significantly higher fHb values were found in patients after WRBC transfusion at the end of surgery in the citrate group, we found no differences in fHb after transfusion of WRBC. The amount of fHb measured by Mortelmans however, is higher than that expected after the passive infusion of fHb present in WRBC alone and implies ongoing hemolysis in the citrate group after transfusion of WRBC. Both in our study and in the Mortelmans study, no differences were found in fHb in WBRC. However, Mortelmans did find higher fHb in waste fluids in the citrate group compared to heparin, something which was not part of our protocol. It seems likely that the washing cycle may have masked significant differences in the fHb in the WRBC between study groups. Our findings after transfusion of WRBC gave no indication of ongoing hemolysis differences between groups. Differences in findings could, of course, be due to the differing surgical procedures studied.

Since ACD-A is a hypotonic solution, the osmotic effect on red blood cells would increase with increasing ATBR. However, the ATBR and incubation time of blood in the BCR was comparable in both groups. Consecutive washing cycles could possibly have an impact on WRBC quality but the volume of red blood cells washed twice and the proportion of patients per study group whose red blood cells were washed twice, were again comparable between groups.

During the washing cycle, the centrifuged volume of red blood cells at which the cell saver sensor detects the buffy coat will have been comparable between the groups. In the citrate group because of the larger MCV, there are therefore fewer red blood cells. This can explain the lower Hb, but not the lower Ht, in the citrate group (since the MCH was comparable between the groups). The same cell saver device was used in all patients but can be expected to deliver a comparable Ht of WRBC in both groups, only when MCV is similar. A larger MCV, with a comparable MCH, may have caused a less efficient centrifugal movement of RBC in the citrate groups during washing. This may have led to a more dilute WRBC, which would also explain why the volume of WRBC in the citrate group was higher (though not significantly), although the total volume of fluid in the BCR was comparable between the groups. Since the product of Hb and the volume of WRBC was comparable between the study groups, the total amount of autologous Hb transfused in both groups was comparable, which argues against significant differences in hemolysis between groups. Fortunately, the quality guidelines for perioperatively salvaged RBCs, published by the American Association of Blood Banks (AABB), were met in both groups (Hct > 50%, Hb > 15 g/dl, fHb < 1.0 g/dl) [14]

There was a significantly lower residual thrombocyte count in the WRBC in the heparin group. This could be an effect of more pronounced thrombocyte activation in the heparin group. Both in vitro [8] and in vivo [16], platelet degranulation, measured by release platelet factor 4 (PF4) is increased by heparin compared to citrate, and therefore most likely a Ca-dependent process, which is likely downregulated by the chelating effect on free calcium of citrate. It seems plausible that platelet activation by heparin and subsequent aggregation may cause a higher proportion of platelets to be washed out during the washing cycle, causing lower thrombocyte counts in the primary infusion bag of washed erythrocytes. A more dilute red blood cell concentrate in the citrate group may allow thrombocytes to infiltrate between red blood cells, leading to a higher thrombocyte count in WRBC. The smaller thrombocyte volumes may explain why only the thrombocyte count and not the total WBC count, was higher in the citrate group. At present, the clinical implications of this finding remain unclear. However, low platelet count has routinely been described after centrifugal cell salvage techniques and recent cell salvage methods utilizing ultrafiltration haven been advocated because they demonstrated improved thrombocyte values in salvaged blood [17].

The inflammatory response elicited by cardiac surgery is characterized by a significant release of both pro- and anti-inflammatory cytokines [18–20] and cell salvage with washing has been demonstrated to ameliorate the inflammatory profile of cardiotomy suction blood [19, 20]. Based on studies mainly performed in the setting of extracorporeal circuits in hemodialysis or hemofiltration, it has been hypothesized that citrate, by causing a decrease is extracellular calcium concentration, effects cytosolic calcium concentrations, which acts as an intracellular messenger [7] activating neutrophils and platelets and subsequent mediator release. Bohler et al. [21], comparing citrate to heparin dialysis, demonstrated (through reduced neutropenia, C3a levels, and lactoferrin release) that depletion of ionized calcium reduced neutrophil degranulation in the extra-corporeal circuit. Two other studies [16, 22] in a similar population found that citrate diminished the release of MPO that is released by (activated) neutrophils. Finally, in a study in ICU patients undergoing CVVH, Tiranathanagul et al. [23], found higher post-filter serum MPO levels in the heparin group compared to citrate. Citrate significantly decreased systemic pre-filter serum MPO and IL-8 levels. To date, no effects of citrate versus heparin have been documented effecting IL-10 levels either in extracorporeal circuits in renal replacement therapy or in cell salvage. Despite the fact that some studies have found no differences when comparing heparin and citrate as anticoagulants in renal replacement therapy [24, 25], there is overwhelming evidence that citrate seems to downregulate inflammation, while heparin seems to be pro-inflammatory at higher concentrations [7], a conclusion reinforced by our findings.

Interpretation of the differences found in WBC differentiation in the washed RBC concentrate is difficult because of minimal baseline differences in WBC differentiation, though the effects of citrate versus heparin as described above, especially the differing effects on polymorphonuclear degranulation, could play a role.

Undoubtedly this pilot study has a number of limitations; primarily it is a small, single center study, despite which findings are statistically robust. The larger anticoagulant priming volume could cause early hemolysis when ATBR was highest, but this was masked by the washing process. A blood smear and quantification of osmotic fragility might have added reinforcing data. Heparin was given systemically in both study groups. Despite equivalent doses in both groups, a supra-additive effect of systemic heparin with heparinized saline in the heparin group cannot be excluded totally. Differences in blood loss during the first 24 h, may have been nullified by the standard algorithm for the treatment of postoperative bleeding and the effect of differing techniques and outcomes between surgeons could be a possible confounding factor. Furthermore, blinding was not complete until the patient left the operating room.

## Conclusion

This study comparing citrate vs. heparin in cell salvage in adults undergoing coronary artery bypass grafting (CABG) found significant differences in the washed red blood cell concentrates: Hb and Hct were lower in the citrate group and the washed red blood cells had a higher MCV and lower MCHC. Residual trombocyte count was higher in the citrate group. Despite these differences, WRBC concentrates in both groups met the quality guidelines published by the American Association of Blood Banks. There were no differences in the necessity for transfusion of allogenic blood products intraoperatively and postoperatively nor in coagulation parameters. Higher IL-10 was found in the citrate group in the BCR, higher myeloperoxidase (MPO) in heparin group patients after WRBC infusion a possible reflection of a proinflammatory effect of heparin compared to citrate anticoagulation.

It has been speculated that intraoperative use of hemo-adsorption therapy may alleviate the hyperinflammatory response triggered by cardiopulmonary bypass during cardiac surgery, though as yet little evidence exists for a decrease in proinflammatory cytokines [26, 27]. A larger randomized controlled study combining hemo-absorption therapy and cell salvage with citrate in the setting of CABG should be undertaken.

## Supplementary Information

**Additional file 1: Supplement 1.** Protocols for Anaesthesia, cardiopulmonary bypass and cell salvage procedure. **Supplement 2.** Baseline laboratory values and postoperative values, before WRBC transfusion, reported as mean (SD) or median (25–75%).

## Data Availability

The datasets during and/or analysed during the current study available from the corresponding author on reasonable request.

## Abbreviations

APTT: Activated partial thromboplastin time
ATBR: Anticoagulant to blood ratio
BCR: Blood collection reservoir
BMI: Body mass index
CABG: Coronary artery bypass grafting
CS: Cell salvage
ECMO: Extracorporeal membrane oxygenation
fHb: Free hemoglobin
Hb: Hemoglobin
Ht: Hematocrit
ICU: Intensive care unit
IL: Interleukin
INR: International normalized ratio
MCH: Mean corpuscular hemoglobin
MCHC: Mean corpuscular hemoglobin concentration
MCV: Mean corpuscular volume
MPO: Myeloperoxidase
PT: Prothrombin time
ROTEM: Rotational thromboelastometry
RDW: Red cell distribution width
WRBC: Washed red blood cell concentrate
TFVCR: Total fluid volume of collection reservoir
WBC: White blood cell
