# Supplementary material for: The effects of differing anticoagulant regimes on blood quality after cell salvage in coronary artery bypass grafting (CABG): a pilot study
Source: J Cardiothorac Surg. 2023 Apr 8;18:116. doi: 10.1186/s13019-023-02246-w (PMC10082980; doi:10.1186/s13019-023-02246-w)
Supplement: Supplementary file 1 — Additional file 1: Supplement 1. Protocols for Anaesthesia, cardiopulmonary bypass and cell salvage procedure. Supplement 2. Baseline laboratory values and postoperative values, before WRBC transfusion, reported as mean (SD) or median (25–75%). [file 13019_2023_2246_MOESM1_ESM.docx]

**Anaesthesia.** All patients were preoperatively sedated with sublingual lormetazepam (0.025 mg/kg) 2 hours before surgery. Diazepam (0.05 mg/kg), sufentanil (0.5 µg/kg) and propofol (1 mg/kg) were used to induce general anaesthesia. Neuromuscular blockade was performed with cisatracurium (0,2 mg/kg) and surgical antibiotic prophylaxis with cefazoline (50 mg/kg, max 2 gr). Tranexamic acid (30 mg/kg) was administered to all patients before incision. Anaesthesia was maintained with sevoflurane or continuous propofol infusion and a continuous infusion of sufentanil (0.25 - 0.5 µg/kg.u). Muscle relaxation was maintained with a continuous infusion of cisatracurium.

**Cardiopulmonary bypass*.*** An identical cardiopulmonary bypass (CPB) circuit (S3 model, Stockert Instruments GmBH, Munich, Germany) was used in all patients. Identical tubing systems were used (Dideco perfusion tubing system Phisio, Sorin group Italia) in the CPB circuits.

Priming volume was a mixture of Plasmalyte (Baxter SA) and Volulyte 6 % (Fresenius Kabi). Pump flow target was 2.4 - 2.8 l/min/m2, blood temperature target 37°C and mean arterial pressures were maintained between 50 - 80 mmHg. The pH-stat principle was applied. Blood from the surgical field was reinfused during CPB.

To prevent intraoperative clotting in patient or CPB circuit, each patient was administered a standardized, weight-adjusted dose of heparin (300 IU/kg), even in the ACD-A group. Maintenance of anticoagulation was achieved with a continuous infusion of heparin (100 IU/kg.hour). Activated clotting time (ACT) target was > 480 seconds. Intermittent doses of heparin were administered by the perfusionist accordingly. After weaning from CPB, heparin was antagonized with protamine in a 1:1 ratio. If ACT was still > 150 seconds, additional doses of protamine were given by the anaesthetist. Any residual CPB volume was sent to the cell saver BCR before starting the first washing cycle.

**Cell Salvage procedure*.*** Cell saver type (Xtra autotransfusion system, Sorin group) was identical in each procedure. Suction pressure was limited to -120 mmHg to minimize hemolysis (24). Blood was filtered with a 40 - 120 µm filter. Cell saver bowl volume was 225 ml. Washing volume was 3 to 4 times bowl volume. Centrifugation speed was 5600 RPM. Normal saline was used as washing fluid. Washed RBC were transfused with a 40 µm filter.

Heparinized saline (25000 IU of heparin [Leo Pharma] per 1 litre normal saline [0.9% NaCl]) was made locally. ACD-A^®^ is commercially available (ACD-A (Fresenius Kabi AG) 22.0 g sodium citrate dehydrate, 24.5 g glucose monohydrate, 8.0 g citric acid monohydrate per litre water ).

Measures were taken to keep (ATBR) as constant as possible. Composition of both anticoagulants enabled an *identical administration rate* of 60 to 80 drops per minute in moderate blood loss, with an ATBR target of 0.2, as prescribed by the UK Cell Salvage Action Group (10). This means blinding of the perfusionist would have been possible, but cumbersome and would have added minimum extra quality to the study design.

Perfusionists adjusted administration to increasing blood loss, but adjustments were limited by *priming* the cell saver BCR and its filter with 300 ml of anticoagulant, before surgical incision.

Cell saver *suction was stopped* before starting the first washing cycle, to maintain ATBR that had been reached at that time. Volumes were tracked to *calculate ATBR* (Total Fluid Volume in BCR [TFVCR], AntiCoagulant Volume [ACV], waste fluid and Washing fluid) (Figure 1).

ATBR is not the only factor possibly affecting washed RBC quality. That is why *total fluid amount and type* administered by the anaesthetist and perfusionist intra-operatively was tracked as well as the *volume of RBC that was washed twice*.

**Supplement 1. Protocols for Anaesthesia, cardiopulmonary bypass and cell salvage procedure**

| **Baseline** | General (n= 38) | Citrate (n=19) | Heparin(n=19) | P-value |
| --- | --- | --- | --- | --- |
| Creatinine (mg/dL) | 0.98 (0.88-1.08) | 0.91 (0.83-1.02) | 0.99 (0.94-1.11) | 0.065 |
| eGFR (ml/min/1.73 m2) | 82 (70-89) | 83(72-90) | 77 (62-83) | 0.065 |
| Ureum (mg/dL) | 36 (28.0-40.3) | 33 (26-38)) | 38(34-48) | 0.103 |
| CRP (mg/L) | 1.5 (0.8-4.6) | 1.3 (0.8-4.6) | 1.6 (0.8-4.6) | 0.954 |
| Hb (g/dl) | 14.7 (13.9-15.7) | 14.7 (14.1-15.7) | 14.7 (13.5-15.8) | 0.583 |
| Ht (%) | 43.5 (40.5-46.6) | 43.3 (41.0-46.3) | 43.7 (39.8-47.6) | 0.708 |
| RBC (*106/ml) | 4.7 (4.5-5.2) | 4.7 (4.4-5.2) | 4.8 (4.5-5.2) | 0.665 |
| MCV (fL) | 90.9 (4.6) | 91.9 (4.7) | 90.1 (4.4) | 0.248 |
| MCH (pg) | 30.8 (1.6) | 31.2 (1.3) | 30.4 (1.7) | 0.161 |
| MCHC (g/dl) | 33.8 (0.86) | 33.9 (1.03) | 33,7 (0.72) | 0.598 |
| RDW (%) | 13.3 (13.0-13.9) | 13.4 (13.0-13.6) | 13.3 (13.0-14.3) | 0.630 |
| Thrombocytes | 217.0 (179.5-270.3) | 217.0 (184.0-263.0) | 201.0 (175.0-274.0) | 0.488 |
| WBC (*1000/ml) | 7.4 (6.7-9.1) | 8.0 (7.1-9.1) | 7.3 (5.7-8.2) | 0.092 |
| APTT (s) | 30.6 (27.7-32.2) | 31.1 (27.5-31.7) | 30.3 (28.0-32.4) | 0.578 |
| Pt (%) | 91 (7) | 93 (5) | 90 (9) | 0.180 |
| INR | 1.04 (1.01-1.10) | 1.04 (1.01-1.08) | 1.05 (1.01-1.13) | 0.201 |
| FHb (mg/dL) | 7.0 (4.8-9.0) | 7.0 (5.0-9.0) | 7.0 (4.0-9.0) | 0.954 |
| Iron (μg/dL) | 83.5 (72.0-113.8) | 83.0 (72.0-113.0) | 84,0 (63.0-116.0) | 0.931 |
| Transferrin (g/l) | 2.1 (1.9-2.4) | 2.0 (1.9-2.4) | 2.1 (1.9-2.4) | 1.000 |
| Ferritin (μg/l) | 199.3 (130.5) | 192.4 (128.7) | 206.3 (135.4) | 0.747 |
| Haptoglobin (g/l) | 1.4 (0.6) | 1.4 (0.5) | 1.5 (0.7) | 0.665 |
| IL-6 (pg/ml) | 3.6 (2.7-6.7) | 3.5 (2.7-6.6) | 5.2 (2.7-7.78) | 0.509 |
| IL-8 (pg/ml) | 15.6 (15.6-15.6) | 15.6 (15.6-15.6) | 15.6 (15.6-15.6) | 0.789 |
| IL-10 (pg/ml) | 3.1 (3.1-6.6) | 3.1 (3.1-5.5) | 3.1 (3.1-9.3) | 0.817 |
| MPO (ng/ml) | 37.4 (29.6-44.8) | 32.3 (27.7-44.5) | 39.3 (32.7-49.0) | 0.229 |
| Hepcidin(ng/ml) | 9.0 (4.7-19.5) | 8.5 (4.6-17.8) | 10.6 (4.7-25.7) | 0.736 |
| WBC (*1000/ml) | 7.4 (6.7-9.1) | 8.0 (7.1-9.1) | 7.3 (5.7-8.2) | 0.092 |
| neutrophils % | 65.08 (9.81) | 62.65 (12.06) | 67.26 (6.88) | 0.162 |
| lymphocytes% | 23.47 (8.74) | 25.88 (11.06) | 21.32 (5.43) | 0.136 |
| monocytes % | 8.0 (7.0-10.0) | 8.0 (7.0-9.5) | 8.0 (7.0-11.0) | 0.731 |
| eosinophils % | 1.0 (1.0-3.0) | 1.0 (0.6-3.5) | 1.0 (1.0-2.0) | 1.00 |
| basophils % | 1.0 (1.0-1.0) | 1.0 (1.0-1.0) | 1.0 (1.0-1.0) | 0.452 |
| neutrophils (*1000/ml) | 4.8 (3.8-6.4) | 4.6 (4.0-6.4) | 5.0 (3.7-6.3) | 0.900 |
| Lymphocytes (*1000/ml) | 1.6 (1.3-2.2) | 1.9 (1.5-3.2) | 1.4 (1.3-1.8) | ***0.006*** |
| Monocytes (*1000/ml) | 0.6 (0.5-0.8) | 0.6 (0.5-0.9) | 0.6 (0.5-0.7) | 0.452 |
| Eosinophils (*1000/ml) | 0.1 (0.1-0.2) | 0.2 (0.1-0.3) | 0.1 (0.0-0.2) | 0.138 |
| Basophils (*1000/ml) | 0.1 (0.0-0.1) | 0.1 (0.0-0.1) | 0.0 (0.0-0.1) | 0.271 |
| cholesterol (mg/dl) | 150 (45) | 148 (47) | 152 (44) | 0.599 |
| non HDL cholesterol (mg/dl) | 108 (39) | 106 (37) | 111 (42) | 0.673 |
| HDL cholesterol (mg/dl) | 42 (14) | 43 (18) | 41 (9) | 0.741 |
| LDL cholesterol (mg/dl) | 77 (35) | 76 (32) | 78 (38) | 0.869 |
| triglycerides (mg/dl) | 156 (69) | 146 (68) | 167 (71) | 0.343 |

| Before WRBC transfusion | General | Citrate | Heparin | P-value |
| --- | --- | --- | --- | --- |
| Fhb (mg/dL) | 26.1 (14.1) | 24.6 (13.7) | 27.6 (14.7) | 0.513 |
| Iron (μg/dL) | 83.0 (70.8-103.5) | 82.0 (61.0-102.0) | 92.0 (78.0-105.0) | 0.418 |
| Transferrin (g/l) | 1.3 (1.2-1.6) | 1.35 (1.2-1.6) | 1.2 (1.18 -1.5) | 0.424 |
| Ferritin (μg/l) | 236.0 (151.2) | 212.9 (125.9) | 259.0 (173.3) | 0.355 |
| Haptoglobin (g/l) | 0.62 (0.33-0.85) | 0.63 (0.29-0.85) | 0.60 (0.36-0.89) | 0.950 |
| INR | 1.40 (0.12) | 1.38 (0.13) | 1.42 (0.11) | 0.222 |
| Pt (%) | 59.49 (7.13) | 61.17 (7.92) | 57.89 (6.07) | 0.166 |
| APTT (s) | 33.9 (30.5-36.1) | 32.9 (30.2-35.1) | 34.3 (31.0-37.0) | 0,234 |
| IL-6 (pg/ml) | 474.0 (289.0-601.0) | 414.0 (208.5-574.0) | 504.0 (292.0-832.3) | 0.287 |
| IL-8 (pg/ml) | 22.9 (15.6-39.3) | 24.2 (16.6-30.5) | 21.2 (15.6-46.9) | 0.732 |
| IL-10 (pg/ml) | 112.7 (56.2-177.7) | 133.0 (69.6-174.2) | 76.1 (33.4-223.5) | 0.287 |
| MPO (ng/ml) | 153.5 (107.8-227.7) | 113.6 (96.7-194.9) | 174.4 (131.2-251.0) | 0.062 |
| Hepcidin(ng/ml) | 22.5 (10.3-32.2) | 15.0 (8.2-30.4) | 24.8 (11.5-33.1) | 0.358 |

**Supplement 2. Baseline laboratory values and postoperative values, before WRBC transfusion,** reported as mean (SD) or median (25-75%).
